# Migration Studies and Endocrine Disrupting Activities: Chemical Safety of Cosmetic Plastic Packaging

**DOI:** 10.3390/polym15194009

**Published:** 2023-10-06

**Authors:** Elias Bou-Maroun, Laurence Dahbi, Laurence Dujourdy, Pierre-Jacques Ferret, Marie-Christine Chagnon

**Affiliations:** 1 PAM UMR A 02.102, Food and Microbiological Processes, Institut Agro, Université Bourgogne Franche-Comté, 1 Esplanade Erasme, F-21000 Dijon, France; 2 Derttech “Packtox”, NUTOX, INSERM U1231, Université Bourgogne Franche-Comté, F-21000 Dijon, France; laurence.dahbi@u-bourgogne.fr (L.D.); marie-christine.chagnon@u-bourgogne.fr (M.-C.C.); 3 Institut Agro Dijon, Service d’Appui à la Recherche, F-21000 Dijon, France; laurence.dujourdy@agrosupdijon.fr; 4 Safety Assessment Department, Pierre Fabre Dermo-Cosmétique, 3 Avenue Hubert Curien, 31035 Toulouse, France; pierre-jacques.ferret@pierre-fabre.com

**Keywords:** NIAS, bioassays, cosmetics, GC-MS, packaging, hERα and hAR reporter gene assays, chemometric, EDC, ED

## Abstract

The endocrine activity and endocrine disruptor (ED) chemical profiles of eleven plastic packaging materials covering five major polymer types (3PET, 1HDPE, 4LDPE, 2 PP, and 1SAN) were investigated using in vitro cell-based reporter-gene assays and a non-targeted chemical analysis using gas chromatography coupled to mass spectrometry (GC-MS). To mimic cosmetic contact, six simulants (acidic, alkaline, neutral water, ethanol 30%, glycerin, and paraffin) were used in migration assays performed by filling the packaging with simulant. After 1 month at 50 °C, simulants were concentrated by Solid Phase Extraction (SPE) or Liquid-Liquid Extraction (LLE). The migration profiles of seven major endocrine disrupting chemicals detected from GC-MS in the different materials and simulants were compared with Estrogen Receptor (ER) and Androgen Receptor (AR) activities. With low extraction of ED chemicals in aqueous simulants, no endocrine activities were recorded in the leachates. Paraffin was shown to be the most extracting simulant of antiandrogenic chemicals, while glycerin has estrogenic activities. Overall, ED chemical migration in paraffin was correlated with hormonal activity. The NIAS 2,4-di-tert-butyl phenol and 7,9-di-tert-butyl1-oxaspiro (4,5) deca-6,9-diene-2,8-dione were two major ED chemicals present in all polymers (principally in PP and PE) and in the highest quantity in paraffin simulant. The use of glycerin and liquid paraffin as cosmetic product simulants was demonstrated to be relevant and complementary for the safety assessment of released compounds with endocrine activities in this integrated strategy combining bioassays and analytical chemistry approaches.

## 1. Introduction

Cosmetic plastic packaging materials are a source of human exposure to many chemicals, including endocrine disruptors (ED). EDs are able to mimic the body’s natural hormones and interfere with the endocrine system by disrupting natural signaling, production, metabolism, transport, and secretion of hormones, contributing to a wide range of adverse health effects. Most of the time, plastics used for packaging contain additives referred to as intentionally added substances (IAS), such as monomers, plasticizers, ultraviolet (UV) absorbers, antioxidants, or lubricants [1]. Because additives are not physically bound to the polymer matrix, they have the ability to migrate from the container to the contents [2,3]. Leaching can involve the migration of additives as well as non-intentionally added substances, often referred to as NIAS [4]. They originate from diverse sources, including impurities in the starting raw materials (monomers, resin), as well as from chemical reactions and degradation products occurring during the manufacturing processes or during transport, storage, and use [5]. In food contact materials, the numbers and levels of NIAS are often significantly higher than what is generally reported for IAS [6,7]. Since they are NIAS and often unpredictable in nature, they are typically not included in databases, and commercial standards are frequently unavailable. This situation makes the identification and confirmation process challenging.

Complex and ambiguously composed mixtures pose difficult challenges in terms of characterization and introduce various hurdles in the context of risk assessment. The scope of direct chemical analysis is constrained due to the extensive array of potentially co-existing chemicals and the inadequacy of suitable analytical techniques for many of them. Additionally, chemical analysis fails to account for possible interactions among individual chemicals within the mixture, which could result in either heightened (additivity, potentiation, or synergism) or diminished (antagonism) biological effects. In this regard, bioanalytical approaches such as in vitro bioassays emerge as promising screening tools capable of identifying a broad spectrum of contaminants based on their biological effects rather than relying solely on their chemical compositions [8,9]. When applied concurrently with chemical analysis, these methods can unearth “unknown” hazardous substances, subsequently allowing for their identification and assessment [10,11].

Materials that come into contact with various substances, such as food or cosmetics, can undergo extraction using various organic solvents, simulating a worst-case scenario of human exposure. Migration tests involving materials aim to evaluate human exposure in a more realistic context, considering that not all the chemicals identified during extraction will be released. These studies can involve migration tests conducted with simulants after they have been in contact with the packaging material [12]. Simulants are uncomplicated matrices employed to replicate interactions with the final product. When it comes to food products, the utilization of simulants is regulated by EC 10/2011 [13], which pertains to plastic materials and articles designed for use with food.

Over the past few decades, there has been a growing focus on safety concerns related to cosmetic products. In Europe, the regulation of cosmetics is governed by EC 1223/2009 [14]. However, in contrast to Food Contact Materials (FCM), there is no specific legislation regarding materials used in cosmetic contact. Indeed, cosmetic contact can vary significantly from food contact, and as of now, there are no specific regulatory simulants [15], although some have been recommended. Indeed, in the case of cosmetic products, simulants like water, ethanol, glycerin, or paraffin were employed by Murat et al. [16,17] to examine the migration of specific phthalates. A more comprehensive investigation could be undertaken to identify other released substances of toxicological concern. Therefore, it is of utmost importance to develop strategies to address this issue and improve the accuracy of mimicking exposure.

The objective of this study was to formulate a strategy for assessing the toxicological impact of chemicals in cosmetics with respect to their hormonal activities when they come into contact with plastic packaging. In this work, migrations of chemicals from 11 materials into 6 different simulants were assessed using GC-MS after SPE or LLE sample preparations. The data treatment was based on multivariate statistical analysis.

In this study, the interactions of leached chemicals in respective extract samples with androgen and estrogen receptors were investigated using gene reporter bioassays. These bioassays are recommended for identifying in vitro estrogenic or androgenic substances (level 2 of the conceptual framework regarding the mode of action of endocrine disruption, OECD, 2018). Estrogen Androgen Thyroid Steroidogenesis (EATS) is one of the criteria adopted to identify endocrine disruptors for plant protection products and biocides (EU) no 528/2012 and (EC) no 1107/2009; guidance No 528/2012 and (EC) No 1107/2009).

## 2. Materials and Methods

### 2.1. Chemicals and Materials (Selected Packaging)

Eagles Minimum Essential Medium (EMEM) without phenol red, Leibovitz’s L-15 medium without phenol red, and phosphate-buffered saline (PBS) were from Gibco (Fischer Scientific, Illkrich, France). 17β-estradiol (E_2,_ CAS: 50-28-2, E2758), ICI 182,780 (fulvestrant, CAS: 129453-61-8, I4409), 5α-dihydrotestosterone (DHT, CAS:521-18-6, D5027), and hydroxyflutamide (CAS:52806-53-8, H4166) were from Sigma-Aldrich (Saint-Quentin Fallavier, France). Fetal bovine serum (FBS) and charcoal-dextran stripped FBS (CD/FBS), 0.25% (*w*/*v*) trypsin/1 mM EDTA solution were from PAN, Biotech (Dutscher, Brumath, France). 7,9-di-tert-butyl1-oxaspiro (4,5)deca-6,9-diene-2,8-dione (CAS 82304-66-3, ≥99.5%) was purchased from LGC (Augsburg, Germany). 

For simulants, glycerin (99.5%) came from Oleon (Ertvelde, Belgium) and liquid paraffin (referred to here as paraffin) from Esso—SAF Exxon Mobil (Notre Dame de Gravenchon, France). Ethanol (96%) was purchased from Crislalco (Chateaubriand, France). Citric acid (≥99.5%) was purchased from Sigma-Aldrich (St. Quentin Fallavier, France). Disodium hydrogen phosphate dihydrate (≥99.5%) and sodium hydroxide (1 M) were obtained from VWR (Fontenay sous Bois, France). Purified water was produced by a Merck Millipore Milli-Q system.

Eleven common cosmetic packaging materials (M) selected from different European suppliers were used in the study. They were made up of polyethylene terephthalate (PET; M1 and M2), mix of PET and recycled polyethylene terephthalate (50% PET/50% rPET; M3), polypropylene (PP; M4 and M5), styrene acrylonitrile copolymer (SAN; M6), and mix of polyethylene (PE): M7 in high-density PE (100% HDPE), M8 in linear low-density PE and cross-linked low-density PE (70% LLDPE/30% XLDPE), M9 and M10 in coextruded LLDPE and XLDPE with ethylene vinyl alcohol COEX 70% LLDPE/30%XLDPE//EVOH), and M11 in HDPE and LLDPE (70%HDPE/30%LLDPE). 

Three materials, namely 100% PET, 100% PP, and COEX 70% LLDPE/30% XLDPE, were acquired from two different suppliers to enable a comparison of material variations from different sources. Details of each item are provided in Table 1. The surface area in contact with the product was determined based on the shape, and this surface corresponds to the area in contact with the cosmetic formula in a commercially available product.

### 2.2. Migration Tests

Selected simulants were put into contact with the different packaging. According to the standard ISO/TR 18811:2018 [18], packaging filled with simulants was kept for 1 month at 50 °C. This experimental condition permitted an accelerated aging process for the products. Then, the simulants were emptied into inert glass containers to stop any further migration. The samples were kept at 4 °C until analysis. Blanks were prepared by introducing simulants in a glass container. In total, six simulants were studied: S1 to S6 (Table 2).

### 2.3. Extraction of Simulants and Sample Preparations

**Figure 1:**
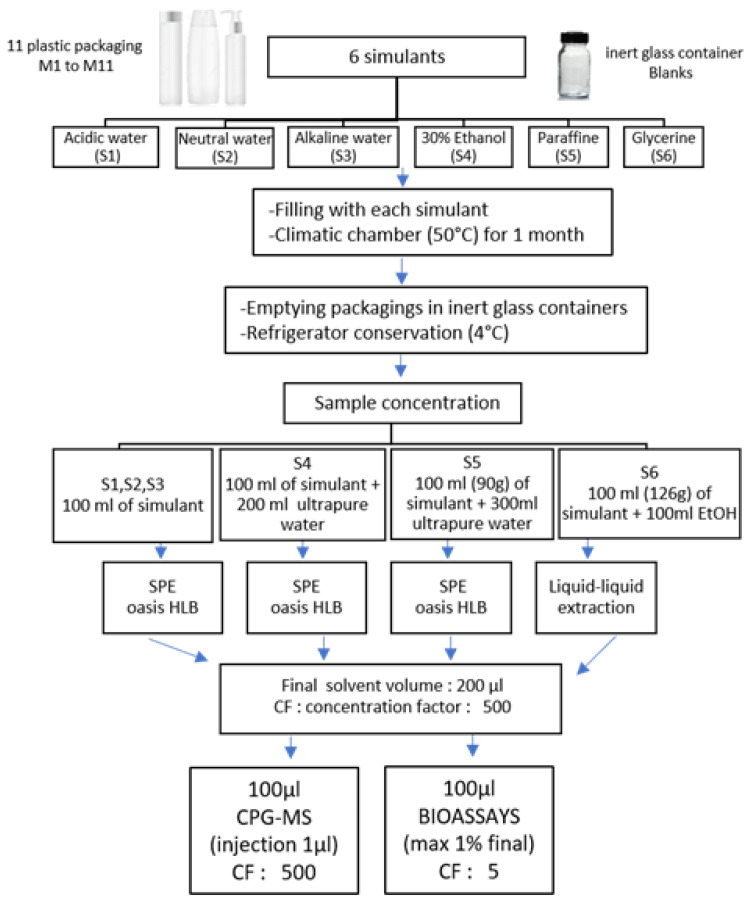
Extraction and sample preparation procedure for all simulants. CF = Concentration Factor.

The extraction procedure is described in Figure 1.

#### 2.3.1. Solid Phase Extraction (SPE)

Oasis HLB glass cartridges (200 mg/5 cc, Waters) were used for SPE. Conditioning of the cartridges was performed with 5 mL of methanol, followed by 5 mL of ultrapure water. After loading each sample at a flowrate of 15 mL/min, the cartridges were rinsed with 5 mL of ultrapure water and dried under vacuum for 60 min. The elution was carried out with 2 × 5 mL of methanol in a glass vial. Methanol was removed under a gentle stream of nitrogen at 40 °C, yielding a final extract of 200 µL, which was separated in two. One 100 µL for bioassays and 100 µL for analytical GC-MS. In order to determine the hormonal activity, the SPE extracts were passaged in 100 µL of DMSO solvent. Then, extracts were stored at −20 °C before analysis. A sample concentration factor of 500 was reached at the end of the SPE procedure.

#### 2.3.2. Liquid/Liquid Extraction (LLE)

Since paraffin is neither soluble in water nor in common alcohols, an LLE procedure was performed. To 100 mL (90 g by weight) of paraffin were added 100 mL of ethanol (96%). The extraction was made by shaking a magnetic stirrer (for 4 h). Then, the decanted ethanolic phase was evaporated using a rotary evaporator (40 °C, 70 mBar) until a final volume of approximately 5 mL. This latter volume was finally evaporated under a gentle stream of nitrogen at 40 °C to a final extract volume of 200 µL. A concentration factor of 500 was reached at the end of the LLE procedure.

### 2.4. ER and AR Transactivation Assays (TA)

The ER transactivation assay using hERα-HeLa-9903 cells was performed according to the OECD test guideline TG455 [19]. The AR transactivation assay using MDA-kb2 cells, originally described by Wilson et al. [20], was performed essentially according to the protocol modified by Ermler et al. [21] and with the method described in the OECD TG458 [22] for the results analysis and interpretation.

#### 2.4.1. Cell Culture 

For the cell-based ER-mediated bioassay, stably-transfected hERα-HeLa-9903 cells were obtained from the Japanese Collection of Research Bioresources (JCRB-N°1318) cell bank. These cells contain stable expression constructs for human ERα and firefly luciferase, respectively. The latter is under the transcriptional control of five ERE promoter elements from the vitellogenin gene. hERα-Hela9903 were maintained in Eagles Minimum Essential Medium (EMEM) without phenol red, supplemented with kanamycin (60 mg/L) and 10% (*v*/*v*) fetal bovine serum in an incubator under 5% CO_2_ at 37 °C. MDA-kb2 cells were derived from the MDA-MB-453 breast cancer cell line and stably transfected with the murine mammalian tumor virus (MMTV)-luciferase.neo reporter gene constructs were obtained from the American Type Culture Collection (Manassas, VA, USA) (ATCC, N°. CRL-2713). Cells were routinely maintained in Leibowitz-15 (L-15) medium supplemented with 10% (*v*/*v*) fetal bovine serum at 37 °C without CO_2_. The two cells were sub-cultured when confluent over a maximum of 10 passages.

#### 2.4.2. Assessment of Cell Viability

At the end of the 20 to 24 h exposure period, cell viability was measured in dedicated plates using the RealTime-Glo MT assay kit (Promega, Madison, WI, USA) according to the manufacturer’s instructions for the end-point method. Briefly, 100 µL of treatment was removed from each test well and replaced by 50 µL of 2× MT cell viability substrate and NanoLuc enzyme diluted in assay medium. Plates were then incubated at 37 °C for 20 min, followed by a well-by-well luminescence measurement in a Chameleon luminometer (from Hidex, Turku, Finland). The light produced was quantified in relative light units (RLUs) and normalized to the average response in the solvent control wells (set to 100%). Only concentrations inducing at least 80% viability were analyzed.

#### 2.4.3. Transcriptional Assays

Prior to experiments, cells were acclimated for at least two media changes in an experimental medium composed of L-15 (MDA-kb2) or EMEM (Hela) medium without phenol red supplemented with 10% charcoal-dextran-treated fetal bovine serum. Cells were then plated in clear bottom white luminometer 96-well plates at a density of 1 × 10^4^ cells/well in 100 μL experimental medium and allowed to attach for 24 h. Cells were also identically plated in 96-well plates dedicated to the RealTime-Glo MT cell viability assay (Promega, Madison, WI, USA). At the end of the attachment period, 3×-concentrated dosing solutions (50 µL) were directly added in triplicate to the wells of both plates. Plates were briefly agitated on a plate shaker and then returned to the incubator for 20 to 24 h. After the exposure period, luciferase activity was measured in the 96-well plates. Plates were emptied, and DMEM without phenol red and Steady-GloLuciferase Assay System reagent (Promega, Madison, WI, USA) were added to the wells. Plates were agitated for 10 min at 500 rpm on an orbital plate shaker, and luminescence was measured using a luminometer (Chameleon). Luminescence signals were expressed in Relative Light Units (RLU).

For each compound, two independent agonist and antagonist assays were performed, as described below. In the agonist assays, the cells were exposed to the dilution series of the tested chemicals or to the positive control E_2_ (1 nM) or DHT (10 nM) and to the solvent controls (SC). In the antagonist assays, dosing solutions were prepared in an experimental medium supplemented with 0.25 nM DHT (Ermler et al. [21]) or 0.025 nM E_2_ (OECD TG455, [19]) in order to establish a baseline close to the EC50 value for co-exposure to screen for AR or ER antagonism, respectively. Moreover, a solvent control without DHT or E_2_ was added to 3 wells. Dilution series of DHT (final concentrations from 10^−13^ to 10^−8^ M) or E_2_ (from 10^−14^ to 10^−8^ M) and of the antiandrogen hydroxyflutamide (from 10^−11^ to 10^−6^ M) or the antiestrogen ICI 182,780 (from 10^−10^ to 10^−5^ M) were used as dose response controls to validate the assay for detection of agonistic and antagonistic activities (Figure S1). Non-linear regression analyses were performed in GraphPad Prism version 9.5.1 by fitting the data to the sigmoidal four-parameter Hill equation (Y = bottom + (top−bottom)/(1 + 10 ^(logEC50–X)^ where Y is the response, X is the logarithm of concentration, and bottom and top are fixed to 0% and 100%, respectively, of the maximum achieved response), from which the EC50 or IC50 values were extrapolated. The results of the reference chemicals are within the acceptable range (Table S1).

#### 2.4.4. Treatment Cells

The concentrations of samples were expressed as concentration factors (CF), which can be derived from the enrichment factor of the sample extraction (500×) multiplied by the dilution factor in the bioassay. DMSO extracts were tested at the highest non-cytotoxic concentration of the solvent (1%) in culture medium, yielding a maximum final concentration factor of 5. Each extract was tested by a 2 dilution factor series (between 4 or more different concentrations). A concentration factor of 1 represented the real concentration migration.

#### 2.4.5. Raw Data Analysis and Interpretation

These were performed according to the method described in OECD TG 458 for ARTA and TG 455 for ERTA. Relative transcriptional activity (RTA %) was calculated for each concentration of the chemicals. In agonist assays, RTA was relative to DHT (10 nM or E2 1 nM, set to 100%), and calculated as follows: The mean RLU for the vehicle control (SC wells) was subtracted from all replicates (RLU in the well); each was then divided by the mean of the background-subtracted RLU for the positive control (PC) to normalize the endocrine activities on a scale of 0% (ethanol vehicle) to 100% (DHT 10 nM or E_2_ 1 nM). In antagonist assays, RTA was relative to solvent control with DHT (0.25 nM) or E_2_ (0.025 nM), set to 100%, and calculated as previously. The resulting normalized data indicates the RTA of each individual replicate. The triplicates were then aggregated into the mean RTA and standard deviations (SD). If appropriate, the concentrations inducing an RTA corresponding to 10% and 50% of the DHT 10 nM or E_2_ 1 nM effect (EC10 and EC50) were determined in the agonist assays, and the concentrations inducing 30% and 50% inhibition of transcriptional activity induced by solvent control with DHT or E_2_ (IC30 and IC50) were determined in the antagonist assays. A substance is considered positive in the agonist assay if the EC10 can be calculated in at least 2 of 2 or 3 assays; a substance is considered positive in the antagonist assay if the IC30 can be calculated in at least 2 of 2 or 3 assays.

#### 2.4.6. Assays Acceptance Criteria

For ER agonist assay

- The results of the 4 concurrent reference chemicals (17β-E2, 17α-E2, 17α-methyltestosterone, and corticosterone) included in each experiment fell within the acceptable range (Table S1);
- The mean luciferase activity of the positive control (1 nM E2) was at least 4-fold that of the mean vehicle control on each plate;
- The fold-induction corresponding to EC10 of the concurrent PC was greater than 1 + 2 standard deviations of the fold-induction value of the concurrent vehicle control;
- The variability among raw data triplicates (luminescence intensity data) was minimal (CV less than 20%), indicating a reliable EC10.

For ER antagonist assay

- The results of the reference chemical (ICI 182,780) included in each experiment fell within the acceptable range (Table S1);
- The mean luciferase activity of the spike-in control (0.025 nM E2) was at least 4-fold that of the mean vehicle control on each plate.
- For AR agonist assay
- The results of the reference chemical (DHT) included in each experiment fell within the acceptable range (Table S1);
- The mean luciferase activity of the positive control (10 nM DHT) was at least 6-fold that of the mean vehicle control on each plate;
- The fold-induction corresponding to EC10 of the concurrent PC was greater than 1 + 2 standard deviations of the fold-induction value of the concurrent vehicle control;
- The variability among raw data triplicates (luminescence intensity data) was minimal (CV less than 20%), indicating a reliable EC10.
- For AR antagonist assay
- The results of the reference chemical (OH-FLU) included in each experiment fell within the acceptable range (Table S1);
- The mean luciferase activity of the spike-in control (0.25 nM DHT) was at least 5-fold that of the mean vehicle control on each plate.

### 2.5. Gas Chromatography Coupled to Mass Spectrometry (GC-MS)

An amount of 1 μL of each extract was injected into a split/splitless injector held at 280 °C in splitless mode. A fused-silica capillary column (30 m × 0.32 mm ID × 0.5 μm film thickness) coated with a stationary phase DB-5MS (J&W Scientific, Folsom, CA, USA) was used. Helium was used as a carrier gas at a velocity of 43 cm.s^−1^ in constant flow mode. 

For simulants S1, S2, S3, and S5. The oven temperature program was as follows: The initial oven temperature was 55 °C. It was raised to 320 °C at a rate of 15 °C/min^−1^. Finally, the temperature was maintained at 320 °C for 5 min.

For simulants S4 and S6, the oven temperature program was as follows: The initial oven temperature was 45 °C. It was raised to 320 °C at a rate of 5 °C/min^−1^. Finally, the temperature was maintained at 320 °C for 5 min.

The mass detector was a quadrupole mass spectrometer, an MSD 5973 from Agilent, using electron impact (70 eV) in full scan mode (mass range 29–450 uma). The MS source temperature was 230 °C, and the MS Quad temperature was 190 °C. Enhanced ChemStation software (Agilent Technologies, Santa Clara, CA, USA) was used for data treatment. Compounds were identified by comparing their mass spectra with mass spectra in the Wiley and NIST databases and verified using the linear retention index.

### 2.6. Chemometric Analysis

Multivariate data analyses were used to explore chemical profiles in more depth and to determine the existence of specific groups. The goal of these analyses was to extract the main information from the data and express this information as a set of summary indices called principal components. 

Data analyses were carried out using R (version 4.1.2) [23] and RStudio (version 1.4.1106) [24]. The R packages used and their versions are as follows: fmsb (version 0.7.3) [25], FactoMineR (version 2.4) [26], factoextra (version 1.0.7) [27], corrplot (version 0.9) [28], and ggpubr (version 0.4.0) [29].

## 3. Results

### 3.1. Endocrine Activity

To investigate whether plastics contain estrogen receptor agonist/antagonist or androgen receptor agonist/antagonist substances, migration samples were analyzed in reporter gene assays. Agonist or antagonist activities on hERα or AR were measured in the absence or presence of positive controls, E2 or DHT, respectively. 

Migration blanks were inactive in both bioassays. Thus, there was no contamination during sample extraction and analysis.

#### 3.1.1. ER Gene Reporter Assays

**Figure 2:**
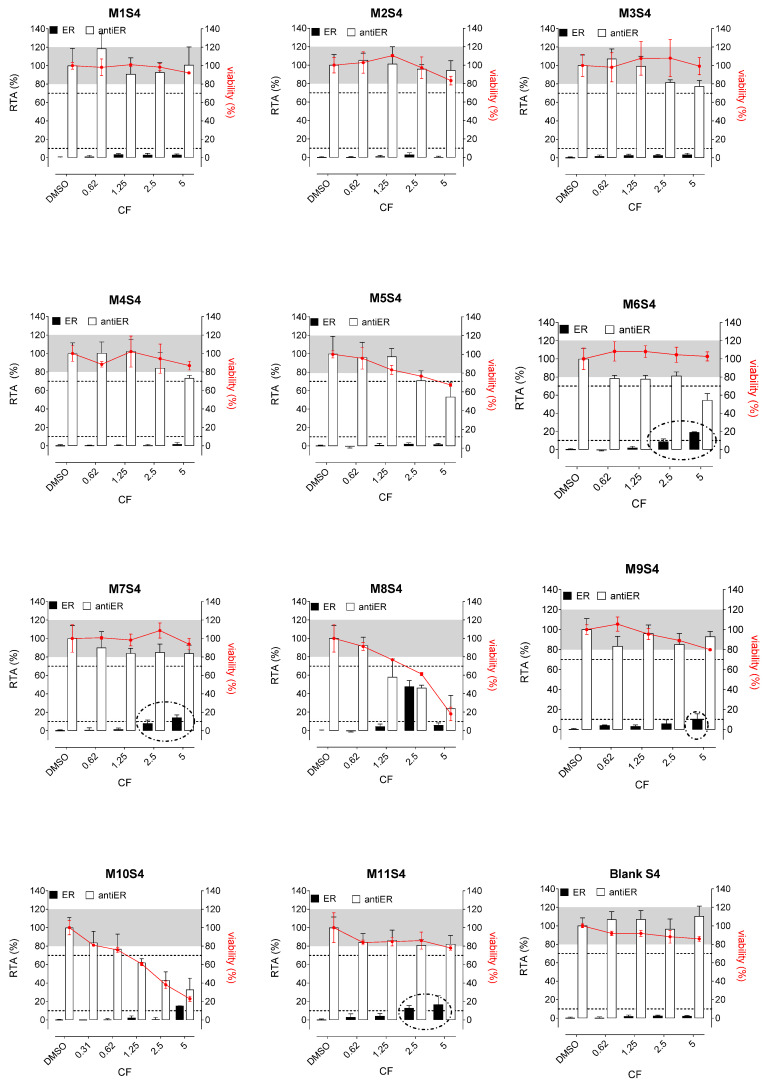
ER agonism and antiER antagonism with packaging extracts (M1 to M11) from the ethanol 30% simulant (S4) in Hela-9903 transcriptional activation assays. Cell viability was evaluated by the RealTime-Glo MT assay. The data represent the mean ± standard deviation of six data points (two experiments each in triplicate). Dotted lines highlight 10% 1 nM E2 normalized relative transcriptional activity (RTA) in the agonist mode or 70% 0.025 nM E2 normalized RTA in the antagonist mode as a threshold for categorizing positive data. E2—17β-estradiol; ER—estrogen receptor; CF—concentration factor.

**Figure 3:**
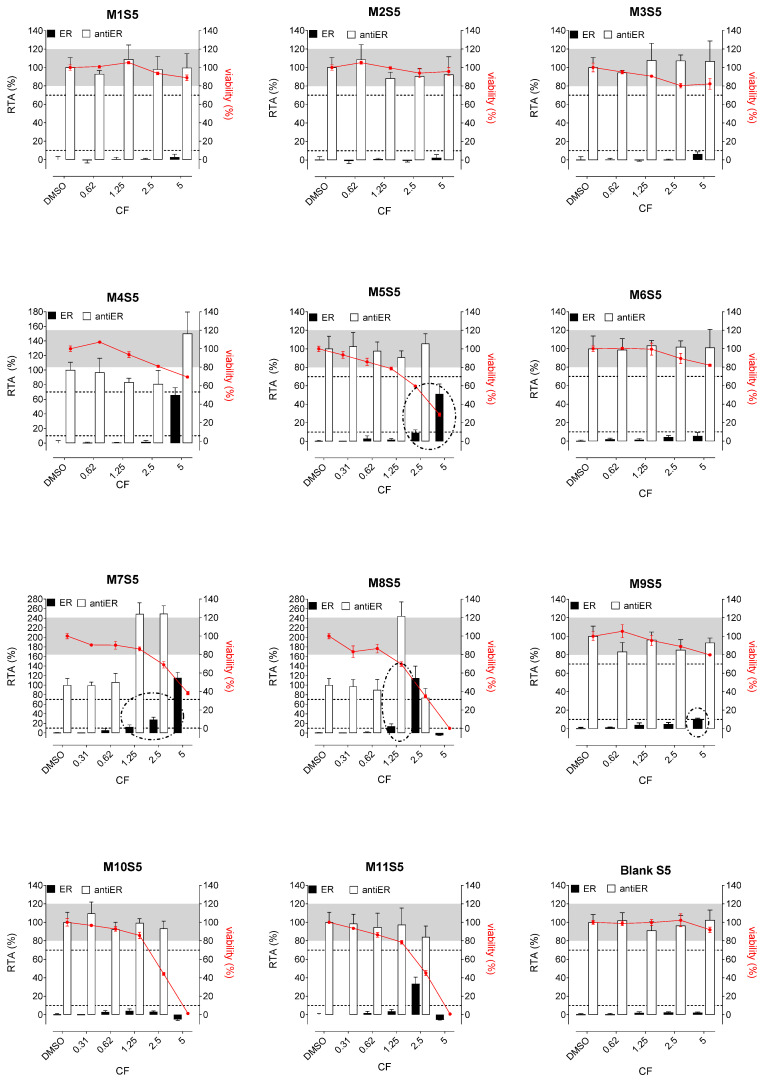
ER agonism and antiER antagonism with packaging extracts (M1 to M11) from the paraffin simulant (S5) in Hela-9903 transcriptional activation assays. Cell viability was evaluated by the RealTime-Glo MT assay. The data represent the mean ± standard deviation of six data points (two experiments each in triplicate). Dotted lines highlight 10% 1 nM E2 normalized relative transcriptional activity (RTA) in the agonist mode or 70% 0.025 nM E2 normalized RTA in the antagonist mode as a threshold for categorizing positive data. E2—17β-estradiol; ER—estrogen receptor; CF—concentration factor.

**Figure 4:**
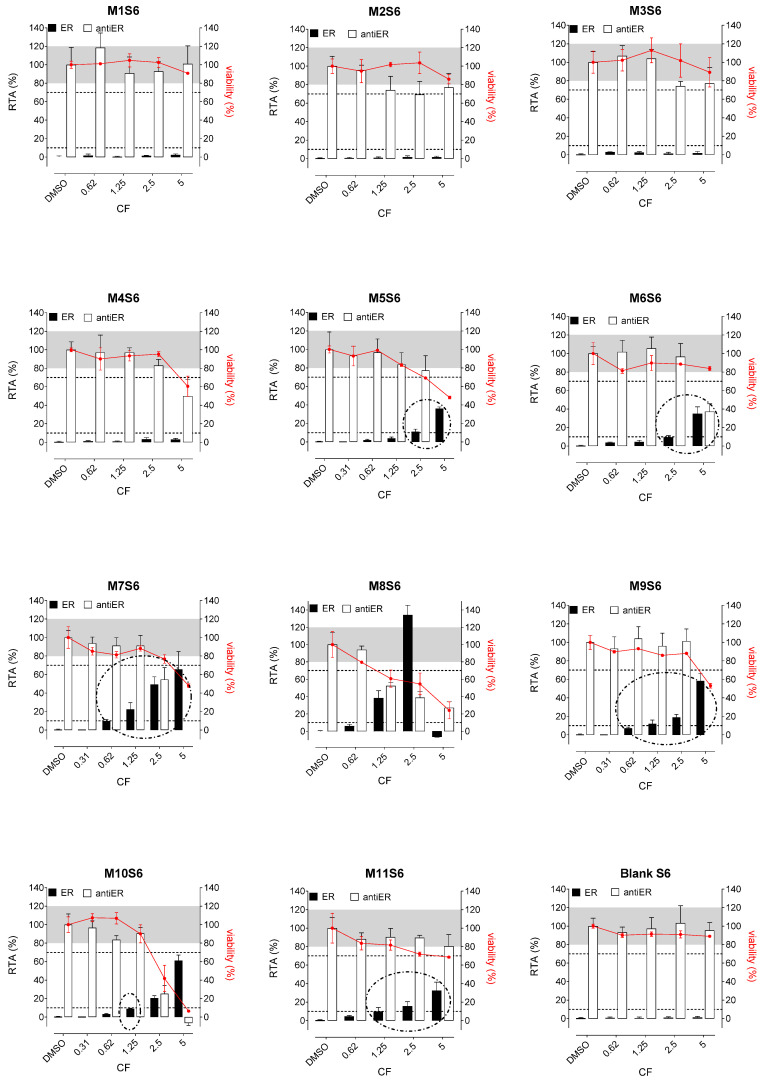
ER agonism and antiER antagonism with packaging extracts (M1 to M11) from the glycerin simulant (S6) in Hela-9903 transcriptional activation assays. Cell viability was evaluated by the RealTime-Glo MT assay. The data represent the mean ± standard deviation of six data points (two experiments each in triplicate). Dotted lines highlight 10% 1 nM E2 normalized relative transcriptional activity (RTA) in the agonist mode or 70% 0.025 nM E2 normalized RTA in the antagonist mode as a threshold for categorizing positive data. E2—17β-estradiol; ER—estrogen receptor; CF—concentration factor.

Whatever the tested extracts, no antiestrogenic activity was observed. In addition, none of the three aqueous simulants or those with PET material (M1, M2, and M3, Figure 2, Figure 3 and Figure 4) were estrogenic. In contrast, extracts from PP (M4 and M5), SAN (M6), and PE (M7 to M11) packaging induced significant ER agonist effects depending on the ethanol or oily nature of the simulants. Glycerin simulant has been shown to be the most effective in extracting estrogenic substance(s), as the activities were high. Indeed, 6/11 extracts (M5, M6, M7, M9, M10 M11) showed a clear dose-response effect (Figure 4), with PE packaging (particularly M7) being the most estrogenic with the highest activity (=50%) at the non-cytotoxic exposure CF = 2.5. Paraffin was the least effective simulant, with only 2/11 migrates (M7 and M9) showing low ER activities at non-cytotoxic concentrations (Figure 3). With ethanol at 30%, an intermediate profile was observed, with 4/11 migrates (M6, M7, M9, and M11) showing low ER activities (10–20%) (Figure 2).

#### 3.1.2. AR Gene Reporter Assays

**Figure 5:**
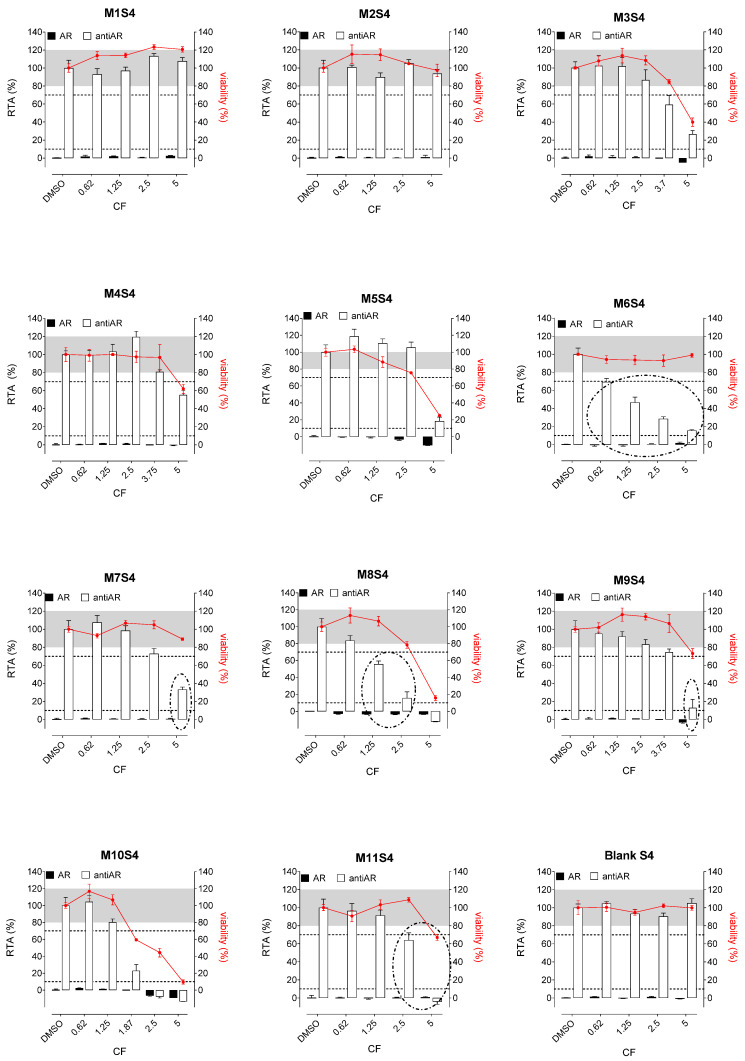
AR agonism and antiAR antagonism with packaging extracts (M1 to M11) from the ethanol 30% simulant (S4) in MDA-kb2 transcriptional activation assays. Cell viability was evaluated by the RealTime-Glo MT assay. The data represent the mean ± standard deviation of six data points (two experiments each in triplicate). Dotted lines highlight 10% 1 nM DHT normalized relative transcriptional activity (RTA) in the agonist mode or 70% 0.25 nM DHT normalized RTA in the antagonist mode as a threshold for categorizing positive data. DHT—5α-dihydrotestosterone; AR—androgen receptor; CF—concentration factor.

**Figure 6:**
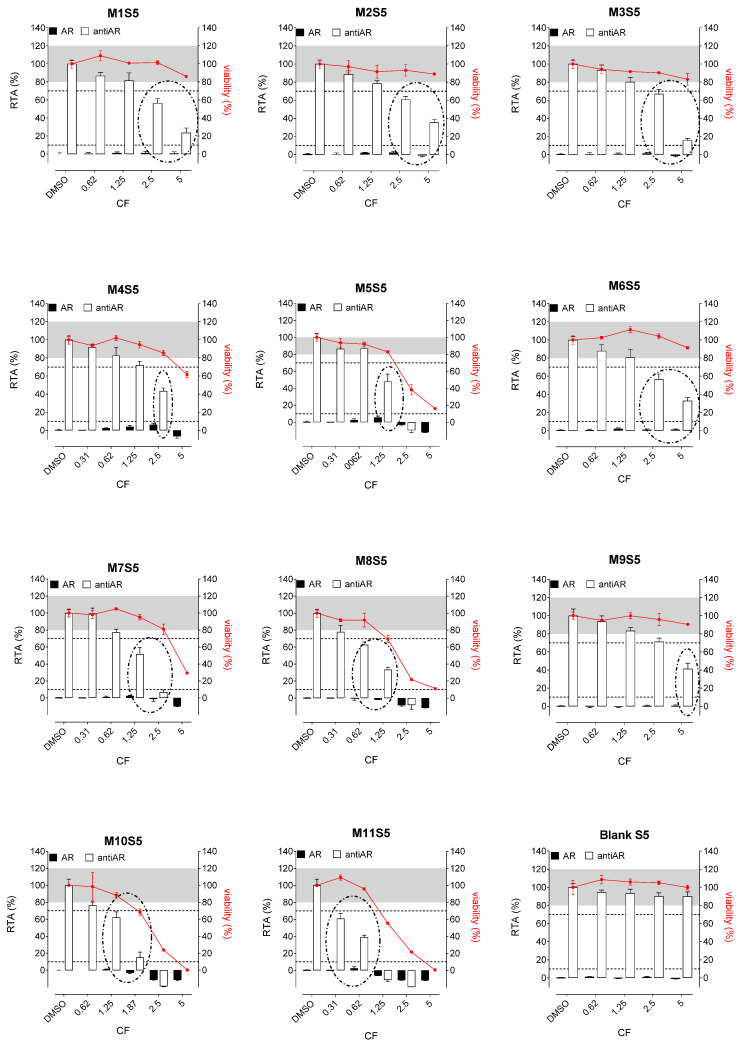
AR agonism and antiAR antagonism with packaging extracts (M1 to M11) from the paraffin simulant (S5) in MDA-kb2 transcriptional activation assays. Cell viability was evaluated by the RealTime-Glo MT assay. The data represent the mean ± standard deviation of six data points (two experiments each in triplicate). Dotted lines highlight 10% 1 nM DHT normalized relative transcriptional activity (RTA) in the agonist mode or 70% 0.25 nM DHT normalized RTA in the antagonist mode as a threshold for categorizing positive data. DHT—5α-dihydrotestosterone; AR—androgen receptor; CF—concentration factor.

**Figure 7:**
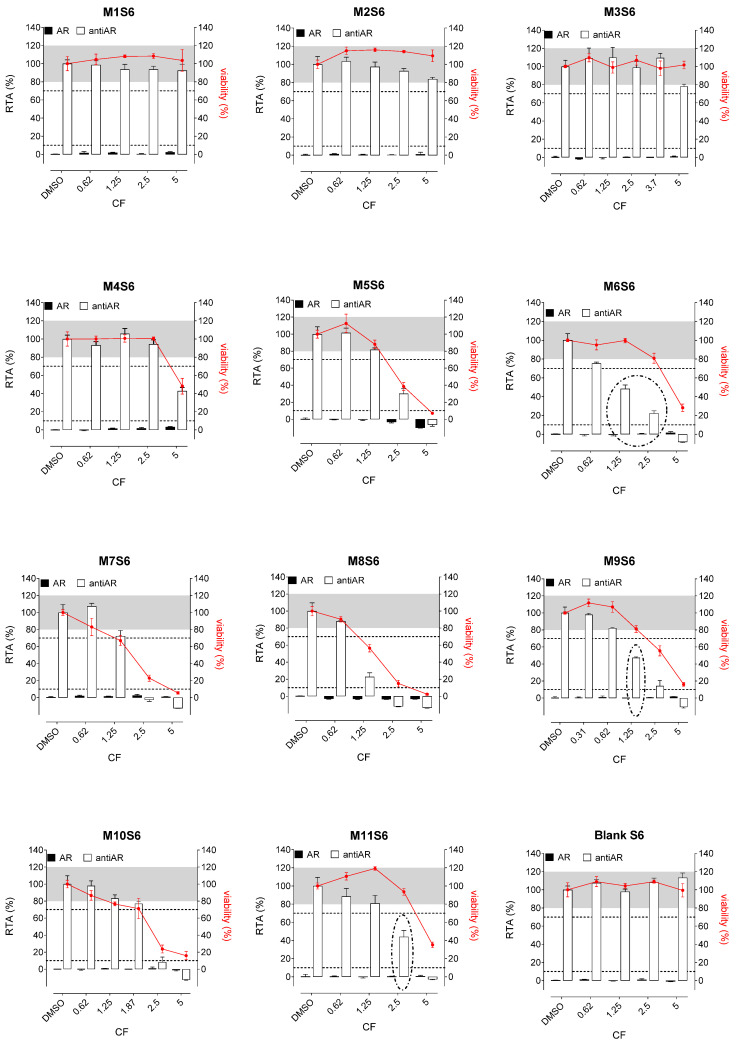
AR agonism and antiAR antagonism with packaging extracts (M1 to M11) from the glycerin simulant (S6) in MDA-kb2 transcriptional activation assays. Cell viability was evaluated by the RealTime-Glo MT assay. The data represent the mean ± standard deviation of six data points (two experiments each in triplicate). Dotted lines highlight 10% 1 nM DHT normalized relative transcriptional activity (RTA) in the agonist mode or 70% 0.25 nM DHT normalized RTA in the antagonist mode as a threshold for categorizing positive data. DHT—5α-dihydrotestosterone; AR—androgen receptor; CF—concentration factor.

Concerning the activities on AR, none of the extracts induced any AR agonist activity in the MDA-kb2 assay (Figure 5, Figure 6 and Figure 7). Furthermore, no antagonistic activity on AR was observed in the three aqueous simulants.

In contrast, paraffin was the most potent simulant in extracting the antiandrogenic substance(s), with 11/11 migrates inhibiting the androgen receptor by 50–90% at non-cytotoxic concentrations (Figure 6).

Glycerin was the less effective simulant, with only 3/11 migrates (M6, M9, and M11) showing antagonistic AR activities at the non-cytotoxic concentrations (Figure 7). Once again, ethanol 30% was an intermediate extracting simulant, with 5/11 migrates (M6, M7, M8, M9, and M11) showing antiAR activities (Figure 5).

### 3.2. Gas Chromatography-Mass Spectrometry 

#### 3.2.1. Non-Targeted Chemical Screening

In this work, 165 chemicals identified in the 66 chromatograms (11 packaging × 6 simulants) were associated with plastics, according to the database “Chemicals associated with Plastic Packaging”, CPPdb, lists A et B Packaging. Among them, 43 chemicals are classified as endocrine disrupting chemicals (EDC) on ER/AR receptors (EDC TEDX list, EDC SIN list, UNEP, Simon et al. [30]. We gave priority to the 7 chemicals that were most frequently identified across all polymers and had the highest abundance in each polymer’s migration (based on peak areas). These identified chemicals were categorized as either intentionally added substances (IAS) or non-intentionally added substances (NIAS).

Table 3 shows the 7 selected most quantitatively present chemicals in the majority of simulants with hormonal activity on ER and/or AR receptors. Except for C2 (7,9-DTBO), hormonal activities were known for the majority of compounds. Then, we tested C2 alone in this study (Figure S2). C2 showed significant antagonistic activity on AR at non-cytotoxic concentrations (Figure S2A). An IC50 of 46 µM was estimated (Figure S2C). Furthermore, it induced a significant ER partial agonistic effect (Figure S2B). An IC50 of 140 µM was estimated (Figure S2D).

#### 3.2.2. Chromatograms

**Figure 8:**
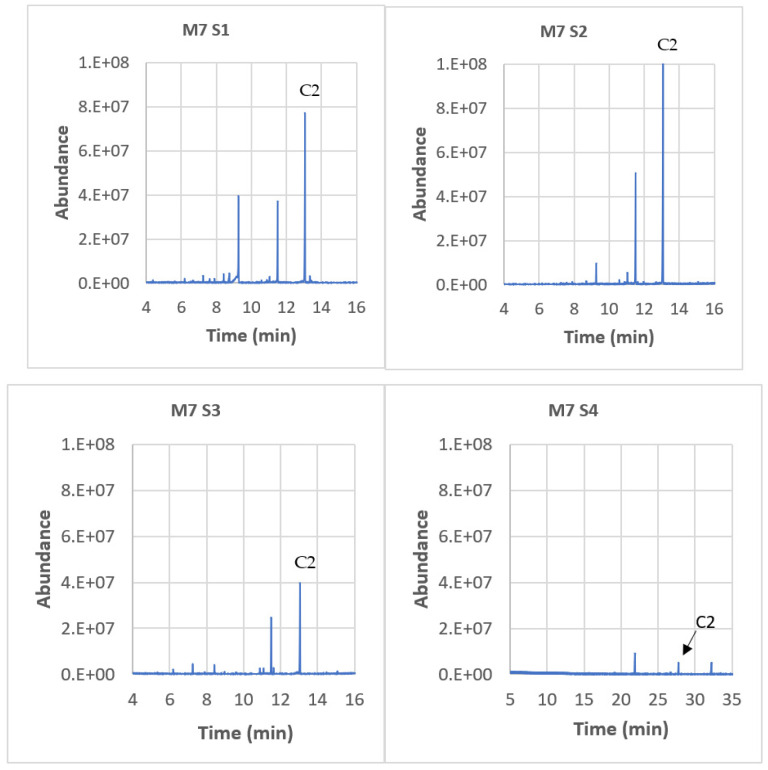

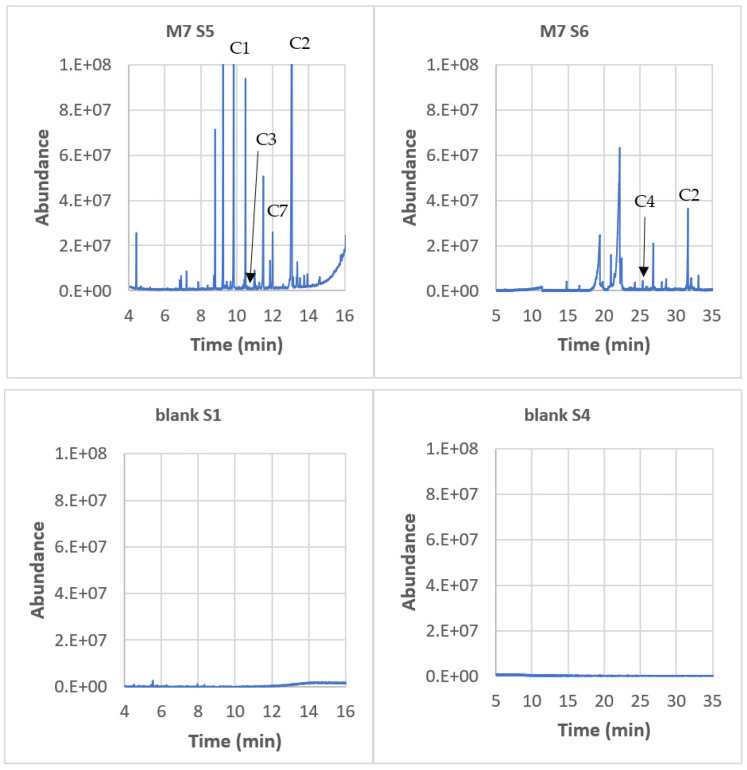
Chromatograms of M7 (HDPE) for all simulants. The blanks are simulants without contact with the material. C1 = 2,4-DTBP, C2 = 7,9-DTBO, C3 = DEP, C4 = BP, C6 = 2-EH, and C7 = 3,5-DTBH. “E” stands for the power of 10.

The chromatogram of Material 7 (HDPE) is presented in Figure 8 for all simulants. Material 7 exhibited the highest release of compounds and was therefore chosen as an example for analysis. Compound C2 (7,9-DTBOD) was always released, regardless of the simulant used. In simulant 5 (paraffin), a significant presence of compounds C1 (2,4-DTBP) and C7 (3,5-DTBH) was observed. Blanks were performed using simulants that had no contact with the materials, and none of the targeted compounds were present. This indicates that the migration of these compounds is really due to contact with the packaging.

**Figure 9:**
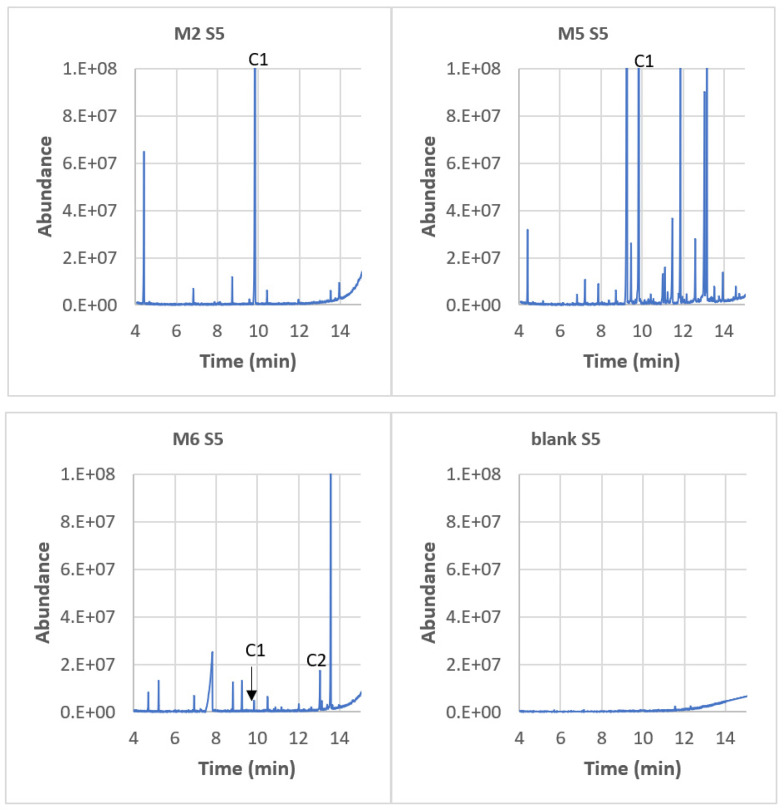
Chromatograms of simulant 5 (paraffin) in contact with M2 (PET), M5 (PP), and M6 (SAN). C1 = 2,4-DTBP, C2 = 7,9-di-tert-butyl1-oxaspiro(4,5)deca-6,9-diene-2,8-dione, C3 = DEP, and C5 = Caprolactam. “E” stands for the power of 10.

S5 (paraffin) is the simulant with the highest number of migrated compounds. It has been selected as an illustrative example (Figure 9). The extraction yield was checked for the worst case: compound C1 in paraffin. C1 was spiked at a concentration of 100 ppb in paraffin, and it was extracted in ethanol. The extraction yield was 105 ± 6 (n = 3 replicates). M2 was chosen as a representative example of a PET-based material, M5 as an example of a PP material, and M6 as an example of a SAN material. In this case, compounds C1 (2,4-DTBP), C2 (7,9-DTBOD), and C3 (DEP) exhibited the highest levels of migration. It is important to note that the blank solution was free of contamination, indicating that the migration originated from the packaging materials themselves.

#### 3.2.3. Migration Profiles

The chemical assessment of plastics is usually made by migration/extraction tests under worst-case conditions using simulants. In order to answer the question: Which simulant provided the worst-case scenario? Chromatogram area values have been normalized by the sum of the areas for each component, expressed as a percentage. Indeed, it gives an account of the amount of the analyzed compound.

**Figure 10:**
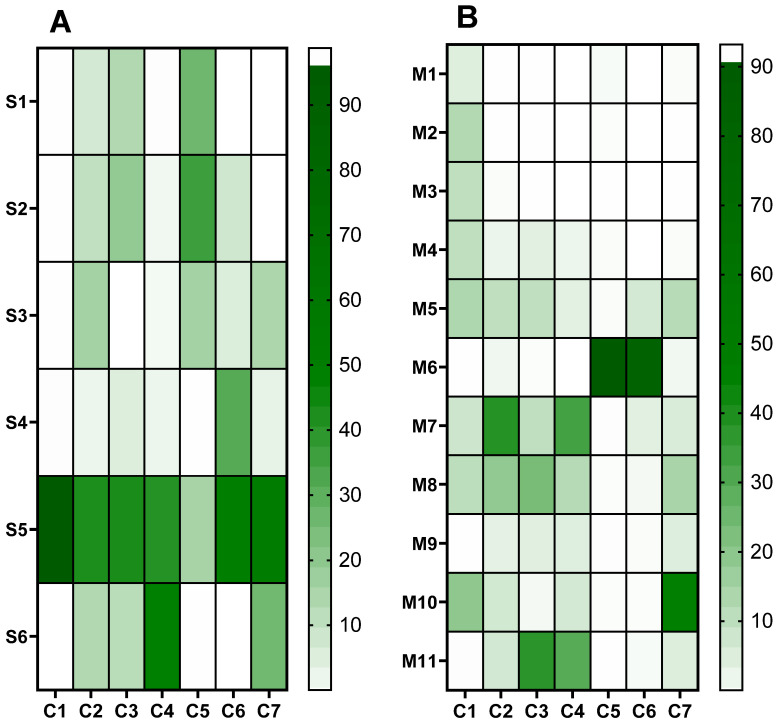
ED chemical profiles for each simulant with all materials (**A**) or for each material with all simulants (**B**). C1 = 2,4-DTBP, C2 = 7,9-DTBO, C3 = DEP, C4 = BP, C5 = Caprolactam, C6 = 2-EH, and C7 = 3,5-DTBH. M1, M2, M3: PET, M4, M5: PP, M6: SAN, M7: HDPE, M8: LDPE, M9, M10: coex LDPE, M11: HDPE/LDPE. S1, S2, S3: water; S4: ethanol 30%; S5: paraffin; S6: glycerin; M: material; S: simulant. Values have been normalized by the sum of the areas for each component, expressed as a percentage.

A heatmap was used (Figure 10A) to visualize the migration profile associated with each simulant. Simulants S1, S2, S3, S4, and S6 (aqueous, ethanol, and glycerin simulants) demonstrated the lowest overall level of chemical migration, except for C5 (S1 et S2) and C4 (S6). S1 and S2 simulants displayed the same profile, with C5 being the most abundant. Simulant 5 (paraffin) exhibited the highest quantities of all compounds (mostly C1) except for C5 (caprolactam) and C4 (benzophenone). Simulant S6 (glycerin) was characterized by the presence of the C4 (BP) and C7 (3,5-DTBHB) compounds and the absence of C1 (2,4-DTBP). Simulant 4 (ethanol 30%) was principally associated with the migration of C6 (2-EH).

The migration profile of each material is visualized in (Figure 10B). C1 (2,4-DTBP) was always present, whatever the polymer, but in the lowest concentration in migrates from M6, M9, and M11. In contrast, C5 (caprolactam) is present only in M6 (SAN). For PET-based materials (M1, M2, and M3), C1 (2,4-DTBP) is the only one to have migrated. With M5 PP-based materials, in addition to C1 (2,4-DTBP), more chemicals were migrated, principally C2 (7,9-DTBO), C3 (DEP), and C7 (3,5-DTBH). Compared to M5, fewer compounds have migrated from M4, which is also a PP polymer. It is worth noting that M6 (SAN) exhibited a distinct behavior compared to other polymers, resulting in the high release of C5 (caprolactam) and C6 (2-ethyl-1-hexanol). In M7 (HDPE), there was significant migration observed from C2 (7,9-DTBO) and C4 (BP) compounds. In M8 (LDPE), C2, C3, and C7 are the three main migrated compounds. M9 and M10 belong to the same family (COEX-LDPE), but their profiles are not equivalent. C1 (2,4-DTBP) is important in M10, whereas C2 is more important in M9. Furthermore, there was significant migration observed from C7 (3,5-DTBH) in M10. M9 and M10 come from two different suppliers and show that the production process could affect the release of chemicals. Finally, M11 (HDPE/LDPE) shows another different profile with the presence of mainly C3 (DEP) and C4 (BP) and, to a lesser extent, C2 and C7.

### 3.3. Multivariate Statistical Analysis 

In order to refine the interpretation, a multiple factor analysis (MFA) was undertaken. MFA [31] represents a multivariate data analysis technique designed to condense and present complex data tables where individuals are characterized by multiple sets of variables, whether they are quantitative or qualitative, organized into groups. It takes into account the influence of all active variable groups when determining the distances between individuals. While the number of variables in each group can vary and the type of variables (qualitative or quantitative) can differ from one group to another, it is essential that variables within a specific group share the same nature [32]. Within the framework of MFA, the simultaneous consideration of multiple sets of variables necessitates the balancing of influences from each set. Consequently, in MFA, there is a weighting process applied to the variables during the analysis. Variables within the same group are normalized using a common weighting value, which may vary from one group to another.

MFA finds applications across diverse fields [33], particularly in scenarios where variables are structured into groups. For example, in survey analysis, where each individual represents a person and each variable corresponds to a question, questions are typically grouped by themes. In our study, 3 groups were considered: the chromatographic group (quantitative), the container group (qualitative), and the hormonal activity group (qualitative). Materials were grouped into families: M1, M2, and M3 were grouped into PET; M4 and M5 were grouped into PP; M6 was grouped into SAN; M7 was grouped into HDPE; M8 was grouped into LDPE; M9 and M10 were grouped into COEX; and M11 was grouped into HDPE/LDPE. Simulants were also grouped into families: aqueous contains S1, S2, and S3; alcohol contains S4. S5 and S6 were grouped into only one family (oil), even if they differed by their polarity properties. S5 was grouped into paraffin and S6 into glycerin.

All data from GC-MS and bioassays were stored in the same table used for all statistical treatments. This table was constructed with 66 rows characterizing packaging materials (M1 to M11) and selected simulants (S1 to S6), and columns corresponding to peak areas of 7 EDs detected by GC-MS (C1 to C7). Area values have been normalized by the sum of the areas for each component, expressed as a percentage. The last column of this table corresponds to the qualitative data obtained from bioassays ER and AR with 2 levels, respectively: the presence (O) or absence (N) of ED activity. 

**Figure 11:**
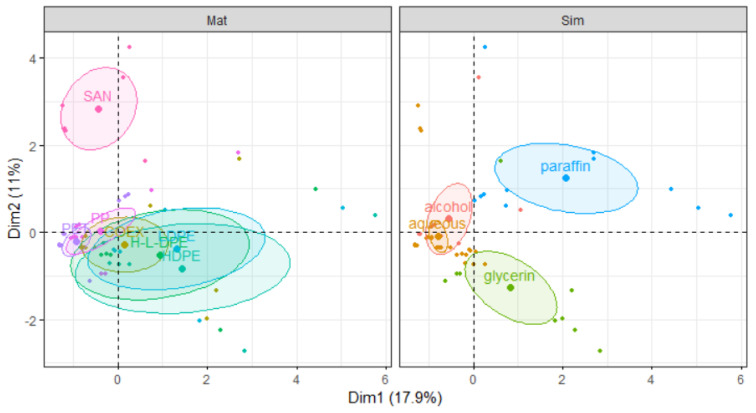
Score plots in the PC1–PC2 plan are grouped by material families on the left and by simulant families on the right.

**Figure 12:**
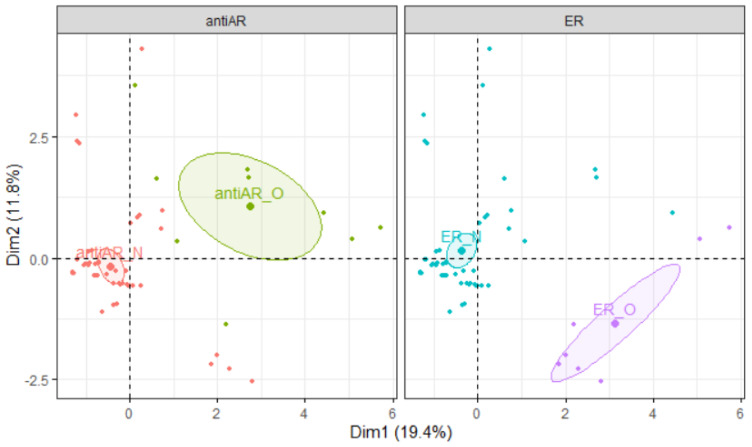
Scores plot in PC1–PC2 plan grouped by antiAR on the left and by ER activity families on the right.

Figure 11 shows the score plot grouped by material and simulant families with 95% confidence ellipses. Individuals with similar profiles are close to each other on the factor map. From the PC1-PC2 plan, it can be observed that there is a good separation between paraffin and glycerin simulants. SAN material is clearly separated from the rest of the materials. Figure 12 shows the score plot grouped by hormonal activities (ER and antiAR) with 95% confidence ellipses. It can be observed that there is a clear separation between the absence (antiAR_N) and the presence (antiAR_O) of antiAR activities measured in the bioassays. The latter was mainly present in the paraffin simulant. However, ER activity was mainly met in the glycerin simulant.

## 4. Discussion 

The objective of this work was to study cosmetic packaging as a source of endocrine-active chemicals and simulate possible migration into different cosmetic formulations.

### 4.1. Migrates of Cosmetic Plastics Packaging Showed In Vitro ER and/or AR Endocrine Activities under Realistic Migration Exposure

A wealth of data exists regarding the endocrine activity of plastics, with a primary focus on Food Contact Materials (FCMs). Previous studies have employed bioassays to evaluate the toxicity leaching from various FCMs [9]. Comparisons conducted using reporter-gene assays have examined the endocrine activity of multiple plastic FCMs, supporting our findings that none of the samples exhibited androgenic effects and that PET (including rPET) did not display estrogenic activities [34,35]. Zimmermann et al. [36,37] noted that the antiandrogenic effects of the migrations from eight major polymer types were more prominent than their estrogenic effects. Similarly, our findings reveal that antiandrogenic activity was more common in PET, PP, SAN, and PE polymers, occurring in 19 out of 66 extracts, compared to estrogenic activity, which was observed in 12 out of 66 extracts. 

In this study, the selection of simulants was based on the anticipated contact scenarios, considering that the materials could potentially come into contact with various cosmetic contents like water, creams, and emulsions. To represent these scenarios, we opted for three distinct aqueous simulants (acidic, neutral, or basic) to simulate oil-in-water emulsions commonly encountered in cosmetics. Additionally, we included an ethanol simulant, specifically chosen for products containing ethanol, such as anti-acne products, fine fragrances, and hair fixatives. We utilized a 30% ethanol concentration to assess the impact of the water/ethanol combination, which closely resembles the proportions typically found in real cosmetic products [16]. Vegetable oils were considered unsuitable simulants for cosmetics. Consequently, we also incorporated glycerin (hydrophilic) and paraffin (hydrophobic) as simulants. These two substances are frequently used as ingredients in various products like lip balms, makeup items, conditioners, and body soaps due to their moisturizing and emollient properties.

No endocrine-disrupting activities were found in any of the water simulants, even at an exposure concentration 5-fold over the migration value CF = 1. A weak anti-androgenicity but no estrogenic activities at the migration value of CF = 1 were observed in ethanol migrates. All aqueous simulants and ethanol (30%) displayed similar low extraction properties, suggesting that active substances are mostly lipophilic.

Moreover, in vitro assessments demonstrated that ER activities were predominantly observed in the glycerin (polar) simulant, whereas antiandrogenic activities were detected in the paraffin (nonpolar) simulant. This aligns with the findings of Creusot et al. [38], showing that ER activities in a multi-contaminated river sediment were mainly due to polar compounds, while antiandrogenic activities were in the less polar fractions. Five samples (comprising 4 PE and 1 PP) exhibited antiandrogenic effects under migration exposure conditions (CF = 1). Specifically, among these samples, only the PE polymer displayed estrogenic activities in the glycerin simulant under realistic migration exposure conditions. Notably, this study represents the inaugural exploration of hormonal activities in paraffin and glycerin when employed as simulants.

### 4.2. Migrates of Cosmetic Plastics Packaging Contain Chemicals Inducing ER and/or AR Endocrine Activities which Are Principally Known NIAS

Non-targeted analyses are used, particularly for food matrices [39,40], and have shown their usefulness in discovering new emerging chemical substances [41]. GC-MS is often used for its sensitivity in food contact material [2,42] or in cosmetic [16,43] studies. Our non-targeted GC-MS results show that cosmetic plastics contain at least 43 chemicals suspected to have endocrine activities via ER/AR receptors. Among them, we have prioritized and quantified (peak areas) the 7 main chemicals from the 11 polymers. 

C1 or 2,4-di-tert-butylphenol (2,4 DTBP), C2 or 7,9-di-tert-butyl1-oxaspiro(4,5)deca-6,9-diene-2,8-dione (7,9 DTBO), and C7 or 3,5-di tert-butyl-4-hydroxybenzaldehyde (3,5 DTBH) were NIAS likely coming from the degradation of antioxidants such as Irgafos 168 for C1 [44] or Irganox 1010 for C2 and C3 [39,45]. 2,4 DTBP was detected as an extractable or leachable NIAS in several studies, associated with PP and PE [46,47], but also with PET and HDPE materials [48,49]. Previous studies found high concentrations of 2,4-DTBP in food packaging materials [41]. Using reporter gene assays, C1 has been shown to have no effect on ER but strong AR antagonist activity [30,50]. Tested in our experimental conditions, it was also antiandrogenic (IC50 = 15 µM) and not estrogenic. Our IC50 value is in the same range (10 µM) as the one found by Satoh et al. [50]. 7,9 DTBO was already identified by García Ibarra et al. [51] in numerous materials. It was discovered in multiple samples from both plastic and paper packaging [52]. Murat et al. [53] adopted a novel thermal extraction method to identify potential leachables from materials utilized in cosmetic packaging. Their study revealed the presence of 2,4 DTBP (except in SAN) and 7,9 DTBO in PP, HDPE, LDPE, and SAN. Our study not only confirmed the presence of these two NIAS in the same packaging materials but also demonstrated their ability to migrate into products such as cosmetic formulas. No data on ED activities for 7,9 DTBO were available, so we tested it in this study. To the best of our knowledge, this marks the initial demonstration of C2 interfering with AR and ER receptors. Comparatively, C2 was 3 times more antiandrogenic (IC50 = 46 µM) than estrogenic (EC50 = 140 µM). Vera et al. [54] identified 3,5 DTBH or C7 as an NIAS that migrates in ethanol 95% and 10% from PP samples. It has been shown to have endocrine-disrupting effects and strong antiAR and weak antiER activities by Simon et al. [30]. 

C3, or Diethylphtalate (DEP), is a common plasticizer that has been used in various industries, including the production of packaging materials. The origin of DEP in packaging materials lies in the intentional addition of this chemical compound during the production process to increase the fluidity of the material. If not bound to the polymer chain, plasticizers may migrate from the plastic into cosmetics [17]. In a paper from Vugt-Lussenburg et al. [55], the authors have cited numerous studies that, in accordance with their own results, showed ER agonism and AR antagonism for DEP.

C4, or Benzophenone (BP), is concerned about its potential to act as an endocrine disruptor with estrogenic activities on the ER receptor. The origin of this compound in tested samples is possibly its use as a UV stabilizer or UV absorber in packaging materials to protect the contents from degradation caused by exposure to ultraviolet (UV) light. BP was shown to interact with the ER receptor using mammalian reporter gene assays [56]. Regarding C6, it was identified by Groh et al. [57] as a compound possibly associated with plastics (database CPPdb, list B). 

C5, also known as Caprolactam, serves as a monomer employed in the production of polycaprolactam (nylon 6). It finds extensive application in the manufacturing of food packaging materials and the production of printing inks [58,59]. It is highly soluble in water and in most common organic solvents (log Kow = 0.66). It is suspected to have ED activities (antiAR and antiER) like those shown with laurolactam [30]. 

Finally, C6, or 2-ethyl-1-hexanol, can be indirectly present in certain packaging materials due to its use as a solvent or additive during the manufacturing process. It is commonly utilized as a solvent for coatings, inks, adhesives, and other materials in packaging production. It can also serve as a plasticizer or a viscosity modifier in certain formulations. C6 was found to be an antagonist of AR and ER [60].

### 4.3. Overall, Endocrine Activities in Paraffin Migrates Are Correlated with ED Chemical Signatures of Polymers

ED activities present in paraffin migrates are well correlated with the chemicals present. C1, or 2,4-DTBP, was the main antiandrogenic NIAS present in all polymers (except in SAN) and largely in paraffin simulant (2,4-DTBP is hydrophobic and nonpolar). In PET, it was the only major EDC detected in S5. In PP, it was associated with C2, and other NIAS have antiAR activity. Finally, in the PE family, the overall anti-AR activity measured in each migrate was associated with more anti-AR chemicals depending of the polymer: C1 or/and C2 were present with C7 in HDPE (M7), C3 in M8 (LDPE) and M11 (HDPE/LDPE), and C7 in M10 (COEX LDPE). On the other hand, in the paraffin simulant, ER activity was correlated with C2, C3, and C4 in the PE group. Among polymer types, only SAN showed a distinct ED chemical signature, with C5 (caprolactam) and C7 (2-ethyl-1-hexanol) as antiAR chemicals.

### 4.4. Estrogenicity Leaching in Glycerin Simulant Was Not Totally Explained by ED Chemical Signatures 

In glycerin, a large part of the observed estrogenicity could be assigned to benzophenone (UV filters), a highly estrogenic compound (EC50 = 5 µM, ToxCast) present in PP and PE (HDPE most) but not in PET and SAN polymers. Furthermore, benzophenone was more extracted by glycerin than paraffin simulant. The highest quantity of BP was found in M7 (HDPE), which was also the most estrogenic. However, in the case of SAN, we have not been able to identify the specific compound responsible in the absence of BP. However, not all major GC peaks in glycerin extracts could be identified. In the future, we will need further investigation regarding the identification of all NIAS. 

To determine the relationship between the 7 main compounds, a Pearson correlation was performed based on peak areas for all simulants and materials combined. C3 (DEP) and C4 (BP) ($\rho_{C3C4}=\rho_{C4C6}=0.61$) were correlated. C1 (2,4-DTBP) and C7 (3,5-DTBH) were correlated ($\rho_{C1C8}=0.58)$. *p*-values were all significant at a risk of 5%. All these correlations could be explained by the close hydrophobicity between the compounds and/or the same polymer origin.

### 4.5. Except for PET, Differences in ED Chemical Signatures between Suppliers Were Observed

Indeed, the chemical fingerprints of the migrates originating from three different suppliers for 3 PET samples were found to be identical, underscoring their specificity for this particular polymer type. Additionally, their profiles of ED activities exhibited similar effectiveness. Conversely, in the case of the two PP and two Coex-LDPE samples, each from two different suppliers within their respective groups, the chemical fingerprints of the migrates displayed more heterogeneity, particularly with the PE polymer. In this scenario, the chemical signature for these two polymers appears to be more specific to the product itself than the polymer type.

### 4.6. Toxicological Aspect

Out of the compounds identified and listed in Table 3, only three of them have specific migration limits specified in European legislation (Regulation 10/2011). Benzophenone, which is possibly carcinogenic to humans [61], is included in Regulation 10/2011 with a Specific Migration Limit (SML) of 0.6 mg/kg. The compound 2-ethyl-1 hexanol is listed with an SML of 30 mg/kg, and Caprolactam is recognized as an authorized additive in plastics with an SML of 15 mg/kg.

One of the most dangerous ED chemicals pinpointed in this investigation was 2,4-DTBP. Liu et al. [62] emphasized that 2,4-DTBP, which had previously received minimal consideration, emerged as the primary synthetic phenolic antioxidant. It contributed to 88.2% and 63.6% of the total target concentrations in the human urine samples before and after undergoing β-glucuronidase hydrolysis, respectively. The elevated concentrations of 2,4-DTBP detected in human urine could indicate the existence of yet-undiscovered pathways of human exposure to this chemical. One potential pathway for human exposure to 2,4-DTBP could be through the consumption of packaged foods. Furthermore, it is worth noting that 2,4-DTBP has also been reported as a natural antioxidant present in rice wine and sweet potatoes [63,64]. Listed on the TEDX list since 2017, C1 is currently on the ECHA CORAP list as a suspected ED. Our study showed that cosmetics can also be one of the routes of 2,4-DTBP exposure to humans.

7,9-DTBO was classified in class III according to Cramer rules. It was also present in all polymers, with the lowest level in PET. 

DEP was the most present in leachates of paraffin from PP, HDPE, LDPE, and almost HDPE/LDPE. The use of certain phthalates, including DEP, has raised concerns regarding their potential impact on human health and the environment. Some studies have suggested that phthalates have adverse effects linked to endocrine disruption. Gopalakrishnan et al. [65] showed that DEP has anti-androgenic activity; it has been associated with adverse reproductive effects in males, but its effects on females have received limited investigation. The estrogen-disrupting activities of DEP remain a topic of debate, necessitating further research to uncover the underlying mechanism responsible for DEP’s estrogenic-disrupting actions. In their study, Fiocchetti et al. [66] assessed the mechanism of action of DEP on the activation status of estrogen receptor alpha (ERα) by examining the receptor’s phosphorylation. Their findings indicated that DEP does not directly bind to ERα. Instead, they propose that DEP engages in multiple parallel interactions with ERα signaling. This underscores the importance of developing an appropriate in vitro method that encompasses the precise molecular mechanisms involved in endocrine disruption.

In this study, we only investigated an in vitro mode of action, which is only one criteria required to identify an endocrine disruptor, as it needs to perform further in vivo experiments to identify an adverse effect and to demonstrate that the observed adverse effect is linked to the identified in vitro mode of action ((EU n°528/2012 and (EC) n° 1107/2009).

Moreover, investigating the mixture effects of detected leakage chemicals and their toxicokinetics in humans is a multifaceted work. Exploring the combined effects of chemicals, comprehending their metabolic pathways within the body, and assessing the potential harm posed by metabolites, particularly in the context of endocrine disruption, could offer valuable insights and prove to be an intriguing area of study.

## 5. Conclusions

This study affirms that exposure to leachates from cosmetic polymers has the potential to impact the endocrine system by modifying the functioning of steroid hormones and disrupting nuclear receptor signaling. Cosmetic Contact Materials (CCM) made from plastics were found to be non-inert, particularly when they came into contact with viscous oily paraffin and glycerin formulations. Upon closer examination of specific migrants, 2,4-DTBP raised significant concern in paraffin migration due to its high detection frequency across all materials as well as its highest concentration levels. Our study stresses the possibility that if one potential human exposure route to 2,4-DTBP is through the consumption of packaged foods, cosmetics could represent another pathway through skin contact.

The insights garnered from this study can contribute to enhancing the risk assessment of various plastic leachates and the development of potentially safer plastic packaging, especially in scenarios where humans may come into contact with cosmetics. Hence, this promising screening approach serves as an initial step in highlighting that glycerin and paraffin act as complementary and effective simulants for identifying unexpected NIAS with hormonal properties. 

## Figures and Tables

**Table 1:** Studied packaging material. PE: polyethylene; PET: PE terephthalate; PP: polypropylene; SAN: styrene acrylonitrile copolymer; rPET: recycled PET; HDPE: high density PE; LLDPE: low linear density PE; XLDPE: cross-linked low-density PE; COEX: coextruded; EVOH: ethylene vinyl alcohol.

Material Code	Material	Filling Volume Appearance	European Supplier	Theorical Surface in Contact with the Cosmetic Product (cm^2^)
M1	100%PET	100 mL clear and colorless elliptical bottle	A	124
M2	100%PET	100 mL clear and colorless cylindrical bottle	B	115
M3	50%PET/50%rPET	200 mL clear and light-yellow elliptical bottle	C	209
M4	100% PP	500 mL opaque and white elliptical bottle	C	362
M5	100% PP	600 mL opaque and dark green cylindrical bottle	B	396
M6	100% SAN	15 mL opaque and white cylindrical bottle	D	63
M7	100% HDPE	100 mL opaque and white cylindrical bottle	E	112
M8	70%LLDPE/30%XLDPE	40 mL opaque and white cylindrical bottle	F	60
M9	COEX 70%LLDPE/30%XLDPE//EVOH	50 mL opaque and white cylindrical tube	F	61
M10	COEX 70%LLDPE/30%XLDPE//EVOH	50 mL opaque and white cylindrical tube	G	66
M11	70%HDPE/30%LLDPE	40 mL opaque and white cylindrical tube	F	59

**Table 2:** Simulants used to mimic the content contact of the different packaging materials.

Nature	Name	Simulants	Justification
Aqueous	S1	Acidic water pH 4 (citric acid and disodium hydrogen phosphate dihydrate)	A large number of cosmetics are oil-in-water emulsions. In these products, the continuous phase exposed to migration is predominantly aqueous. In order to be more accurate, 3 types of water were used.
	S2	Demineralized water pH 7 for neutral water simulant	
	S3	Alkaline water pH 11 (NaOH solution)	
Alcohol	S4	Ethanol 30%	Cosmetics products can contain alcohol (anti-acne, hair-fixative, or fine fragrance products). Water/ethanol 30% is close to real cosmetic products.
Oil	S5	Liquid paraffin	Glycerin (polar) and paraffin (nonpolar) are raw materials commonly used in cosmetic products for their moisturizing and emolliating properties, respectively.
	S6	Glycerin	

**Table 3:** Major GC-MS-identified compounds with in vitro hormonal activities.

GC-MS Identified Compounds	CAS	Name	log Kow	Presence in Material and Simulants	Simulant with the Highest Level
C1	96-76-4	2,4-di-tert-butyl phenol (2,4-DTBP)	5.19	M1 to M11 S1 to S5	S5
C2	82304-66-3	7,9-di-tert-butyl1-oxaspiro(4,5)deca-6,9-diene-2,8-dione (7,9-DTBO)	3.55	M1 to M11 S1 to S6	S5
C3	84-66-2	Diethyl phthalate (DEP)	2.70	M1 to M11 S1 to S6	S5
C4	119-61-9	Benzophenone (BP)	3.18	M4 to M11 S1 to S6	S6
C5	105-60-2	Caprolactam	0.66	M1 to M11 S1 to S6	S2
C6	104-76-7	2-ethyl-1-hexanol (2-EH)	2.73	M5 to M11	S4
C7	1620-98-0	3,5-di-tert-butyl-4-hydroxyl-benzaldehyde (3,5-DTBH)	4.20	M3 to M10 S5 and S6	S5

## Data Availability

The data are available from the corresponding authors.

## Supplementary Materials

The following supporting information can be downloaded at: https://www.mdpi.com/article/10.3390/polym15194009/s1. Figure S1: ER and AR agonism and antagonism with positive controls in Hela-9903 (ER) and MDA-kb2 (AR) transcriptional activation assays. ER, estrogen receptor; AR, androgen receptor; Figure S2: AR antagonism and ER agonism and antagonism with 7,9-di-tert-butyl-1-oxaspiro (4,5)deca-6-9-diene-2,6-dione in MDA-kb2 (AR) and Hela-9903 (ER) transcriptional activation assays. ER, estrogen receptor; AR, androgen receptor (A and B). Hill slope representation to calculate the IC50 variable (C and D). Table S1: Reference Chemicals: performance criteria. Values derived from the concentration response curve (log EC50, log EC10, logIC30, logIC50, Hill slope) for the reference chemicals in the ER agonist assay (A), in the ER antagonist assay (B), in the AR agonist assay (C) and in the AR antagonist assay (D).
