# Supplementary material for: Migration Studies and Endocrine Disrupting Activities: Chemical Safety of Cosmetic Plastic Packaging
Source: Polymers (Basel). 2023 Oct 6;15(19):4009. doi: 10.3390/polym15194009 (PMC10574997; doi:10.3390/polym15194009)
Supplement: Supplementary file 1 [file polymers-15-04009-s001.zip › polymers-2542859-supplementary.pdf]

Supplementary Materials

Migration Studies and Endocrine Disrupting Activities: Chemical Safety of Cosmetic Plastic Packaging

Elias Bou-Maroun [1,*], Laurence Dahbi [2], Laurence Dujourdy [3], Pierre-Jacques Ferret [4], and Marie-Christine Chagnon [2]

[1] PAM UMR A 02.102, Food and Microbiological Processes, Institut Agro, Université Bourgogne Franche-Comté, 1 Esplanade Erasme, F-21000 Dijon, France;

[2] Derttech "Packtox", NUTOX, INSERM U1231, Université Bourgogne Franche-Comté, F-21000 Dijon, France; laurence.dahbi@u-bourgogne.fr (L.D.); marie-christine.chagnon@u-bourgogne.fr (M.-C.C.)

[3] Institut Agro Dijon, Service d'Appui à la Recherche, F-21000 Dijon, France; laurence.dujourdy@agrosupdijon.fr

[4] Safety Assessment Department, Pierre Fabre Dermo-Cosmétique, 3 Avenue Hubert Curien, 31035 Toulouse, France; pierre-jacques.ferret@pierre-fabre.com

* Correspondence: elias.bou-maroun@institut-agro.fr; Tel.: +33-38-077-4080

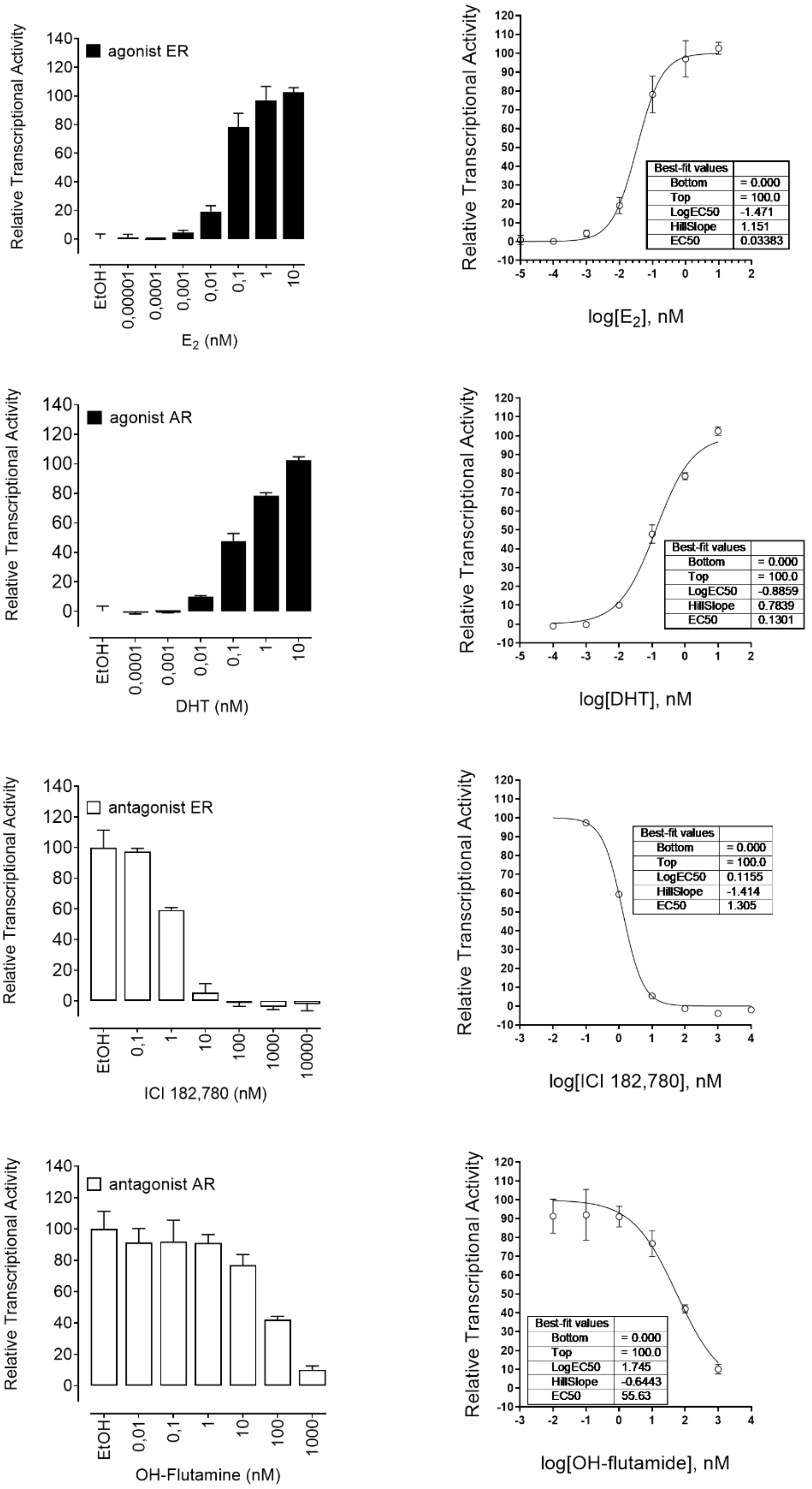

Figure S1. ER and AR agonism and antagonism with positive controls in Hela-9903 (ER) and MDA-kb2 (AR) transcriptional activation assays. ER, estrogen receptor; AR, androgen receptor.

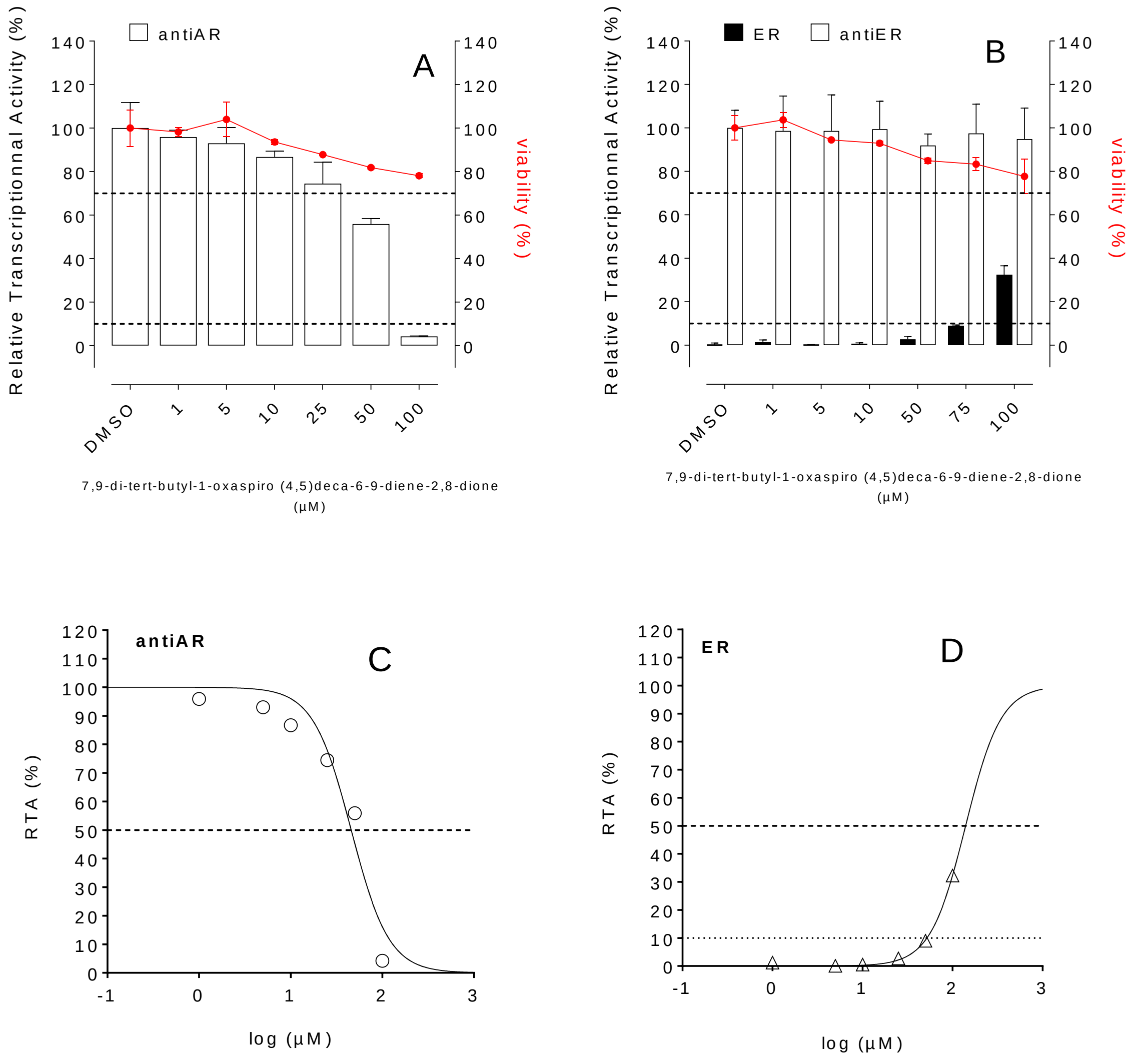

	oxaspiro
Sigmoidal dose-response (variable slope)	
Best-fit values	
Bottom	= 0
Top	= 100
LogEC50	1.66
HillSlope	-2.1
EC50	45.67

	oxaspiro
Sigmoidal dose-response (variable slope)	
Best-fit values	
Bottom	= 0
Top	= 100
LogEC50	2.146
HillSlope	2.215
EC50	139.8

Figure S2. AR antagonism and ER agonism and antagonism with 7,9-di-tert-butyl-1-oxaspiro (4,5)deca-6-9-diene-2,6-dione in MDA-kb2 (AR) and Hela-9903 (ER) transcriptional activation assays. ER, estrogen receptor; AR, androgen receptor (A and B). Hill slope representation to calculate the IC50 variable (C and D).

Table S1. Reference Chemicals: performance criteria. Values derived from the concentration response curve (log EC50, log EC10, logIC30, logIC50, Hill slope) for the reference chemicals in the ER agonist assay (A), in the ER antagonist assay (B), in the AR agonist assay (C) and in the AR antagonist assay (D).

A-Performance Criteria for Reference Chemicals in ER agonist assay

Reference Chemicals Parameter	Acceptable Range (OECD 455)	Value
17β-estradiol (E2)		
log E50 (M)	-11.4 to -10.1	-10.5
log E10 (M)	<-11	-11.3
Hill Slope	0.7 to 1.5	1.15
17α-estradiol		
log EC50 (M)	-9.6 to -8.1	-8.4
log EC10 (M)	-10.7 to -9.3	-9.2
Hill Slope	0.9 to 2	1.14
17α-methyltestosterone		
log EC50 (M)	-6.0 to -5.1	-4.3
log EC10 (M)	-8.0 to -6.2	-6.4
Hill Slope	-	-
Corticosterone		
log EC50 (M)	-	-
log EC10 (M)	-	-
Hill Slope	-	-

B-Performance Criteria for Reference Chemical in ER antagonist assay

Reference Chemical Parameter	Acceptable Range (OECD 455)	Value
ICI 182,780		
log IC50 (M)	-9.8 to -8.2	-8.9
log IC30 (M)	-10.0 to -9.0	-9.1
Flutamide		
log IC50 (M)	-	-
log IC30 (M)	-	-

C-Performance Criteria for Reference Chemical in AR agonist assay

Reference Chemical Parameter	Acceptable Range (OECD 458)	Value
5α-Dehydrotestosterone (DHT)		
log EC50 (M)	-11.03 to -9.00	-9.88
log PC10 (M)	-12.08 to -9.87	-10.48
Hill Slope	0.57 to 4.3	1.79

D-Performance Criteria for Reference Chemical in AR antagonist assay

Reference Chemical Parameter	Acceptable Range (OECD 458)	Value
Hydroxyflutamide (OH-FLU)		
log IC50 (M)	-7.8 to -6.17	-7.25
log IC30 (M)	-8.37 to -6.41	-7.83
